# A Dopamine/Tannic-Acid-Based Co-Deposition Combined with Phytic Acid Modification to Enhance the Anti-Fouling Property of RO Membrane

**DOI:** 10.3390/membranes11050342

**Published:** 2021-05-06

**Authors:** Lixin Xie, Yan Liu, Wen Zhang, Shichang Xu

**Affiliations:** State Key Laboratory of Chemical Engineering, Tianjin Key Laboratory of Membrane Science and Desalination Technology, and School of Chemical Engineering and Technology, Tianjin University, Tianjin 300350, China; xie_lixin@tju.edu.cn (L.X.); liuyan_ly1128@163.com (Y.L.)

**Keywords:** reverse osmosis, anti-fouling, tannic acid, dopamine, phytic acid

## Abstract

Reverse osmosis (RO) membranes are widely used in the field of water treatment. However, there are inevitably various fouling problems during long-term use. Surface engineering of RO membranes, such as hydrophilic modification, has attracted broad attention for improving the anti-fouling performance. In this work, we constructed a green biomimetic composite modification layer on the surface of polyamide membranes using a dopamine (DA)/tannic acid (TA) co-deposited layer to bridge the polyamide surface and hydrophilic phytic acids (PhA). The DA/TA interlayer could firmly adhere to the RO membranes, reducing the aggregation of DA and providing abundant phenolic hydroxyl sites to graft PhA. Meanwhile, the anchored PhA molecule bearing six phosphate groups could effectively improve the superficial hydrophilicity. The membranes were characterized by the SEM, AFM, XPS, water contact angle test, and zeta potential test. After surface modification, the hydrophilicity, smoothness, and surface electronegativity were enhanced obviously. The flux and rejection of the virgin membrane were 76.05 L·m^−2^·h^−1^ and 97.32%, respectively. While the modified D2/T4-PhA membrane showed decent permeability with a water flux of 57.37 L·m^−2^·h^−1^ and a salt rejection of 98.29%. In the dynamic fouling test, the modified RO membranes demonstrated enhanced anti-fouling performance toward serum albumins (BSA), sodium alginates (SA), and dodecyl trimethyl ammonium bromides (DTAB). In addition, the modified membrane showed excellent stability in the 40 h long-term test.

## 1. Introduction

Membrane-based separation technologies, such as ultrafiltration (UF), nanofiltration (NF), and reverse osmosis (RO), have become more and more popular in the industries of seawater desalination and wastewater treatment in recent years [1,2,3]. In particular, RO technology has been applied to various separation and enrichment applications due to its low energy consumption, simple operation, and its environmentally friendly nature [4,5,6]. However, the decrease of permeability, caused by membrane fouling during the long-term use of an RO membrane, needs to be solved urgently [7,8,9,10,11]. Membrane fouling will not only shorten the service life of the RO membrane, but will also increase the operating cost, which will further hinder the widespread application of RO technology [12,13,14]. Generally, the main factors causing membrane fouling are pore blocking, pore constriction, foulant adsorption, and the formation of cake layers [10,15]. It is believed that improving the hydrophilicity of the membrane surface could reduce fouling [7,16,17]. Due to the enhancement in surface hydrophilicity, a hydration layer can be formed on the hydrophilic surface to reduce the adhesion of foulants, thus achieving the purpose of anti-fouling [18].

Inspired by mussels, dopamine (DA) has attracted much attention due to its strong adhesion and simple and mild reaction conditions [19,20,21]. DA can take the reaction of self-polymerization under alkaline conditions and firmly adhere to various surfaces [22]. McCloskey [23] studied the anti-fouling performance of the dopamine-modified RO membranes and found that they exhibited a lower protein adhesion ability than the original membrane. However, long deposition time and easy aggregation are two issues of the surface modification of DA layers [19]. Therefore, efforts are still required to seek out more facile methods to construct efficient DA layers.

Currently, it is worth noting that plant polyphenols, especially tannic acid (TA) and gallic acid (GA), have received extensive attention in membrane modification [24,25,26]. TA exists in many plants and is an inexpensive and easily available polyphenol substance [27] that could solve the problem of DA aggregation. There have also been some related studies on the co-deposition of DA and TA [28,29]. Shao [29] used DA and TA to prepare a more uniform surface coating, which overcame the shortcomings of the DA layer that was easy to aggregate. Under alkaline conditions, the catechol and amino groups of DA can be covalently reacted via a Michael addition/Schiff base reaction. Similarly, the abundant phenolic hydroxyl groups and derivatized quinone groups in polyphenols could also be bound to amino groups of DA through a Michael addition/Schiff base reaction.

Although the polyphenols could provide abundant phenolic hydroxyl groups, the hydrophily of DA/TA layers on the membrane surface is still limited. Compared with the weak acidic phenolic hydroxyl groups, strong phosphonic acids have a higher degree of ionization, showing more affinity toward water molecules. As one of the non-toxic and natural phosphonic acids, phytic acids (PhA) have attracted extensive attention for enhancing the anti-fouling properties of membranes [30,31]. A phytic acid molecule contains six electronegative phosphate groups which can chelate a variety of metal ions; the PhA-metal complex system provides a new strategy for surface modification of membranes [32]. For example, Xiong [30] and Qi [33] constructed PhA-metal complexes through a layer-by-layer self-assembly method to obtain a modified osmosis membrane with fine anti-fouling properties. However, due to the lack of strong complexing sites, it is difficult to introduce the PhA groups onto the membrane surface directly and easily.

Herein, with the combined advantages of DA/TA interlayers and PhA modification, we construct a hydrophilic surface coated on the commercial RO membrane. On the one hand, the co-deposited DA/TA layer could easily and firmly adhere to the RO membrane, while also providing abundant hydroxyl chelating sites to bind PhA. On the other hand, the grafted PhA modification could enhance the hydrophilicity and thus improve the anti-fouling performance of the RO membrane. The surface morphology, chemical composition, surface charge, and hydrophilicity of the membrane had been studied in detail. The permeability was evaluated by 2000 mg·L^−1^ NaCl aqueous solution. The anti-fouling performance was evaluated by three model foulants using bovine serum albumin (BSA), sodium alginate (SA), and dodecyl trimethyl ammonium bromide (DTAB).

## 2. Experimental Section

### 2.1. Materials

The commercial RO membrane LCLE-4040 was from Dow Chemical Company and rinsed with isopropanol and deionized water before use. The chemicals, sodium chloride (NaCl), isopropanol, sodium bisulfite (NaHSO_3_), phytic acid (PhA, ~50% aqueous solution), and hydrochloric acid (HCl) were obtained from Chemart Chemical Technology Co., Ltd. in Tianjin, China. DA, TA, tris(hydroxymethyl) aminomethane (Tris), ferric chloride hexahydrate (FeCl_3_·6H_2_O), as well as serum albumin (BSA) were purchased from Aladdin Bio-Chem Technology Co., Ltd. in Shanghai, China. Sodium alginate (SA) and dodecyl trimethyl ammonium bromide (DTAB) were obtained from Meryer Chemical Technology Co., Ltd. in Shanghai, China. Deionized (DI) water with a conductivity lower than 10 μS/cm was provided by the Distilled Water Business Department of Yongqingyuan in Tianjin, China.

### 2.2. Surface Modification of RO Membranes

**Scheme 1:**
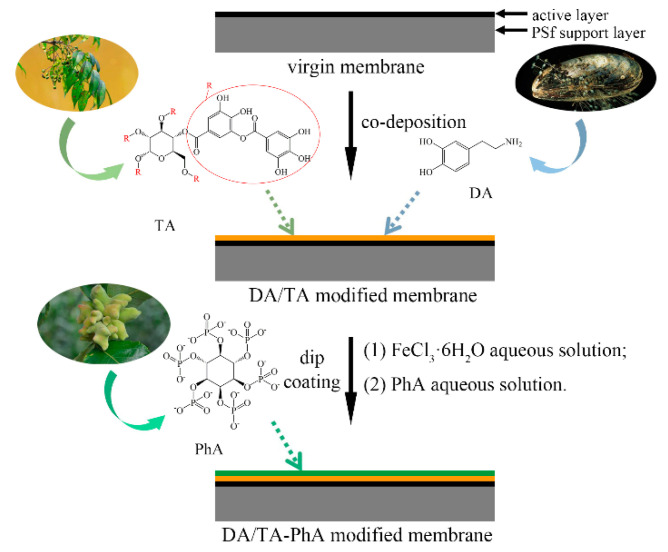
Preparation process of a DA/TA-PhA membrane.

The surface modification of RO membranes was realized through two steps, as shown in Scheme 1.

The first step was the co-deposition of DA and TA. The RO membrane was fixed between the membrane frames (15 cm × 19 cm) to ensure that only the active layer could be reacted with the solution, which was prepared by dissolving different concentrations of DA and TA in Tris buffer (pH = 8.5, 50 mM, 30 mL). In each group of experiments, the concentration of DA was controlled at 2 g/L and the concentration of TA was 0, 2, 4, and 8 g/L, respectively. The reaction was run at room temperature for 1 h. Then, the membrane was taken out and washed with deionized water to get the DA/TA modified membrane. The second step was to use iron ions to load PhA on the membrane, which can be called a DA/TA-PhA modified membrane. The DA/TA-PhA modified membranes were prepared based on the DA/TA modified membranes. Firstly, the DA/TA layer was exposed to 10 mM FeCl_3_ solution (30 mL) for 2 min and then washed with deionized water to remove the excess FeCl_3_. Next, the membrane was contacted with 10 mM PhA solution (30 mL) for 2 min to get the DA/TA-PhA modified membrane. The virgin RO membrane was named as PA-TFC. When the concentration of DA and TA were x and y, respectively, the prepared membrane was named as Dx/Ty-PhA (x = 2 g/L; y = 0, 2, 4, 8 g/L). Before all tests, the prepared membranes were stored in NaHSO_3_ solution.

### 2.3. Membrane Characterization

All membranes were washed with deionized water and dried at 60 °C for 12 h before the characterization. The surface morphology and surface roughness of the membrane were characterized by scanning electron microscopy (SEM, S4800, Hitachi, Japan) and atomic force microscopy (AFM, Dimension Icon, Bruker Co., Bremen, Germany), respectively. The chemical composition of the membrane surface was characterized by X-ray photoelectron spectroscopy (XPS, K-Alpha+, ThermoFisher Scientific, Loughborough, UK) and energy-dispersive X-ray spectroscopy (EDS, NanoSEM430, FEI). The hydrophilicity of the membrane surface was measured by a contact angle goniometer (OCA15EC, Dataphysics, Filderstadt, Germany). The zeta potential was measured by an electro-kinetic analyzer (SurPASS, Anton Paar GmbH, Graz, Austria).

### 2.4. Evaluation of Membrane Permselectivity

The permselectivity was evaluated by a cross-flow filtration device. Before the test, the membranes were pre-compressed with 2000 mg·L^−1^ NaCl aqueous solution at an operating pressure of 1.55 ± 0.1 MPa for 1 h.

The membrane permselectivity was evaluated by the water flux and salt rejection, which were measured using 2000 mg·L^−1^ NaCl aqueous solution under the pressure of 1.55 ± 0.1 MPa at 25 °C. The cross-flow rate was maintained at 1.5 L/min. Besides the temperature, pressure, and cross-flow rate, there are additional factors that also play an important role in RO membrane performance, such as the impact of the feed spacer geometry on the pressure drop and on the mass transfer coefficient related to concentration polarization phenomena [34,35,36]. Here, only the factors of temperature, pressure, and cross-flow rate are considered. The water flux and salt rejection rate were calculated by Equations (1) and (2), respectively.

(1) $$J=\frac{V}{A\cdot \Delta t}$$

where *J* denotes the water flux (L·m^−2^·h^−1^), *V* is the volume of permeated water (L), *A* represents the effective membrane area (3.116 × 10^−3^ m^2^), and Δ*t* is the recorded measurement time (h). Because the RO membrane has a high salt rejection of NaCl, the filtered permeate is generally regarded as pure water (ρ = 1 g/cm^3^). Therefore, the volume of the permeate is obtained by measuring the quality of the permeate in Equation (1).

(2) $$R=(1-\frac{C_{p}}{C_{f}})\times 100\%$$

where *C_p_* and *C_f_* represent the salt concentrations in the permeate and feed, respectively, which are measured by a conductivity meter (DDSJ-308A, Cany precision Instrument Co., Ltd., Shanghai, China).

### 2.5. Membrane Anti-Fouling Property Tests

The anti-fouling performance of the membrane was evaluated by three kinds of model foulants, including the protein (bovine serum albumin, BSA, 100 mg·L^−1^); the polysaccharide (sodium alginate, SA, 100 mg·L^−1^); and the surfactant (dodecyl trimethyl ammonium bromide, DTAB, 50 mg·L^−1^). The membranes were tested under the same initial flux and were evaluated by two filtration-rinsing cycles after pre-compressed in 2000 mg·L^−1^ NaCl aqueous solution for 1 h. One filtration-rinsing cycle included a filtration process for 5 h and a rinsing process for 1 h. The 2000 mg·L^−1^ NaCl aqueous solution was used in the first 30 min of the filtration process. Then, a certain amount of model foulant was added to the NaCl aqueous solution for the anti-fouling test in the next 4.5 h. The flux was recorded by measuring the permeate weight every 5 min. After the filtration process, the feeding solution was completely removed and replaced with deionized water to start rinsing for 1 h. The rinsing process was carried out under the operating conditions of a pressure of 0.7 ± 0.1 MPa and a cross-flow rate of 1.5 L/min. After the two filtration-rinsing cycles, the deionized water in the system was replaced by 2000 mg·L^−1^ NaCl aqueous solution, and the flux was measured again for another 30 min.

The anti-fouling properties of the membrane were characterized by two parameters: the flux recovery ratio (*FRR_i_*, *i* = 1 or 2) and the total flux decline ratio (*DR_t_*). Their calculation formulas are shown as follows:

(3) $$FRR_{i}=\frac{J_{r,i}}{J_{0}}\times 100\%\;(i=1,2)$$

(4) $$DR_{t}=(1-\frac{J_{f}}{J_{0}})\times 100\%$$

where *FRR_i_* represents the flux recovery ratio (%) after the first (*i* = 1) or second (*i* = 2) filtration-rinsing cycles, *J_r_*_,*i*_ is the recovery water flux after the first (*i* = 1) or second (*i* = 2) rinsing (L·m^−2^·h^−1^), *J*_0_ is the initial water flux (L·m^−2^·h^−1^), *DR_t_* denotes the total flux decline ratio (%) after 10 h, and *J_f_* is the water flux measured after 10 h in the filtration test (L·m^−2^·h^−1^).

### 2.6. Stability Assessment

The D2/T4-PhA membrane was chosen as a representative for the stability test. The stability of the D2/T4-PhA membrane was measured under the conditions of 1.55 ± 0.1 MPa and 1.5 L/min using 2000 mg·L^−1^ sodium chloride solution for 40 h. The permeate weight was recorded to calculate the real-time flux. The conductivity of the permeate weight was tested at intervals to calculate the rejection.

## 3. Results and Discussion

### 3.1. Formation Mechanism of Biomimetic Cross-Linked Networks

**Scheme 2:**
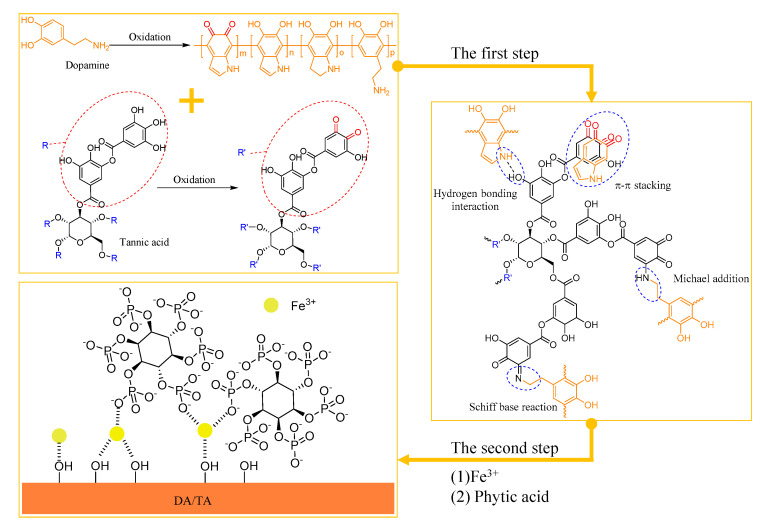
The formation mechanism of a DA/TA-PhA membrane.

The catechol groups contained in DA and TA can be oxidized to quinone groups under alkaline conditions, and the amino groups and phenolic groups in DA can be cross-linked with TA through Michael addition and Schiff base reactions during the DA/TA co-deposition process [29], as shown in Scheme 2. Due to the introduction of TA, more hydroxyl groups were loaded on the surface of the membrane; thus, the iron ions can be successfully fixed on the surface of the membrane by the chelation of catechol groups and metal ions [37]. Then, PhA, containing abundant phosphoric acid groups that could be coordinated with iron ions, were thus easily grafted onto the membrane surface [38].

### 3.2. Surface Morphology Analysis

**Figure 1:**
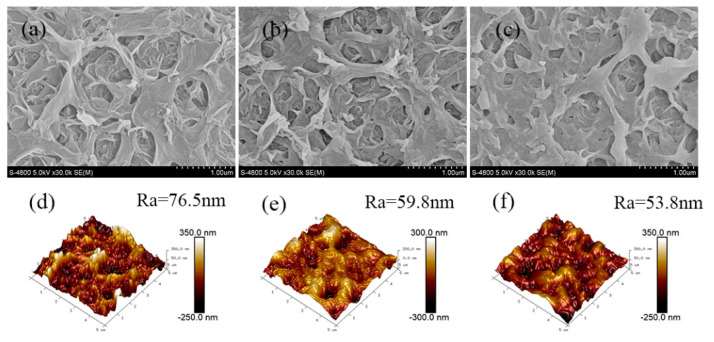
Surface SEM and AFM images of (**a**,**d**) virgin PA-TFC, (**b**,**e**) D2/T4, and (**c**,**f**) D2/T4-PhA membranes.

The morphology of the film surface was characterized by SEM and AFM. From the SEM images in Figure 1a–c, it can be seen that all membranes have the typical ridge-valley structure of a polyamide. From the AFM images in Figure 1d–f, after DA/TA co-deposition and PhA deposition, part of the valley structure was covered. The modified membranes presented an obviously smoother surface; the average roughness (Ra) of the membrane was significantly reduced from the original 76.5 nm to 59.8 nm after DA/TA co-deposition. The Ra was further reduced to 53.8 nm after PhA deposition. The average thickness of the selective layer of the original PA-TFC membrane is about 317 nm. The modified D2/T4-PhA has a thicker selective layer, with an average thickness of about 404 nm. The results are shown in Figure S1.

### 3.3. Surface Chemical Composition Analysis

**Figure 2:**
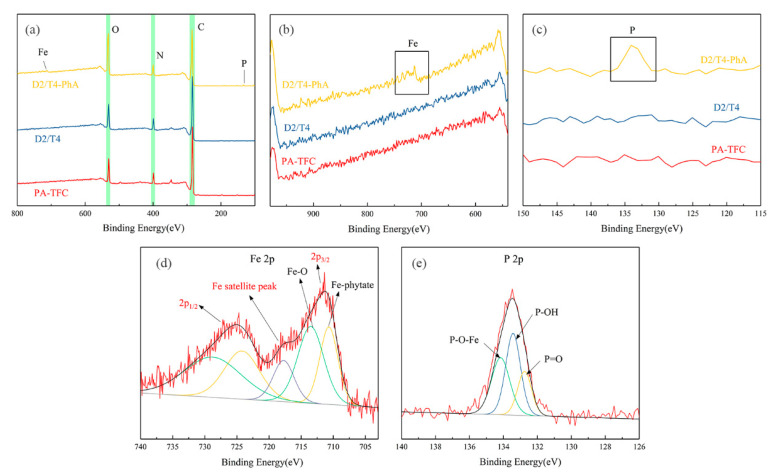
XPS spectra of the virgin and modified RO membranes on (**a**) 800–180 eV, (**b**) 980–540 eV, (**c**) 150–115 eV, and (**d**) Fe 2p or (**e**) P 2p XPS spectra of DA/TA-PhA membrane.

The chemical structure and composition of the RO membrane were analyzed using XPS and EDS. Figure 2a is the full XPS spectra of PA-TFC, D2/T4, and D2/T4-PhA. Compared with the original film, new peaks appeared on the D2/T4-PhA membrane at 134.1 eV and 713.1 eV, representing P and Fe, respectively (Figure 2b,c). In the high-resolution spectra of Fe element (Figure 2d), the peaks at 710.7 eV and 724.2 eV were attributed to Fe^3+^-phytate, and the peaks at 713.6 eV and 729.1 eV were due to the complex of Fe^3+^ and hydroxyl. The peak appearing at 717.7 eV belongs to the satellite peak of the Fe element [38]. From the spectrum of P 2p (Figure 2e), the presence of P = O (132.8 eV), P-OH (133.4 eV), and P-O-Fe (134.2 eV) are presented, confirming that PhA was successfully grafted onto the membrane through the Fe^3+^ complexation [30].

The atomic content on the surface of the membrane was measured by EDS and the results are listed in Table 1. The atomic content of P element in the D2/T0-PhA, D2/T2-PhA, D2/T4-PhA, and D2/T8-PhA membranes were 1.53%, 1.59%, 2.24%, and 1.43%, respectively. With the increase of TA content, TA could provide more active reaction sites to bind the Fe^3+^-PhA complex. However, too much TA could hinder the cyclization and expansion of DA polymer chains, and thus reduce the co-deposition of the DA/TA interlayers. Therefore, the D2/T8-PhA membranes have a decreased amount of complexed Fe^3+^-PhA [29].

### 3.4. Surface Hydrophilicity and Zeta Potential Analysis

**Figure 3:**
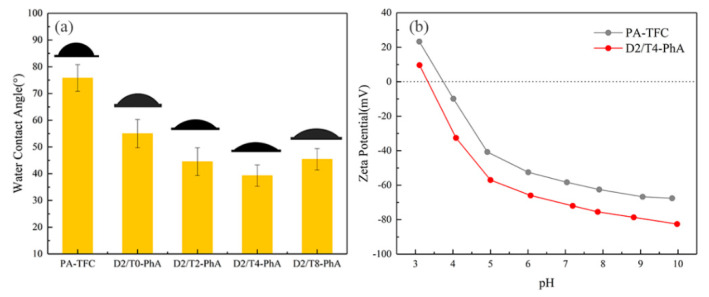
(**a**) Water contact angles and (**b**) zeta potentials of PA-TFC membrane and DA/TA-PhA membranes.

The water contact angles of the membranes were measured to evaluate their surface hydrophilicity. It can be seen from Figure 3a that all modified TFC membranes have lower water contact angles than that of the PA-TFC membrane. In addition, with the increase of TA content, the water contact angle showed a trend of first decreasing and then increasing. The contact angle of the PA-TFC membrane was 75.8°. The contact angle of D2/T4-PhA was the lowest, which was reduced to 39.3°.

The charge properties of the membrane surface were obtained by measuring their streaming potentials in the range of pH 3–10, and the results were shown in Figure 3b. The tested membrane showed amphoteric charge properties. The zeta potential of PA-TFC is 23.3 mV at pH 3 and −67.6 mV at pH 10, while the zeta potential of D2/T4-PhA is 9.6 mV at pH 3 and −82.5 mV at pH 10. Because the hydroxyl and phosphate groups contained in TA and PhA are negatively charged, the membrane surface became more negative after modification [33]. The increase of negative charges on the membrane surface could reduce the negatively charged contaminants by electrostatic repulsion.

### 3.5. Membrane Permselectivity

**Figure 4:**
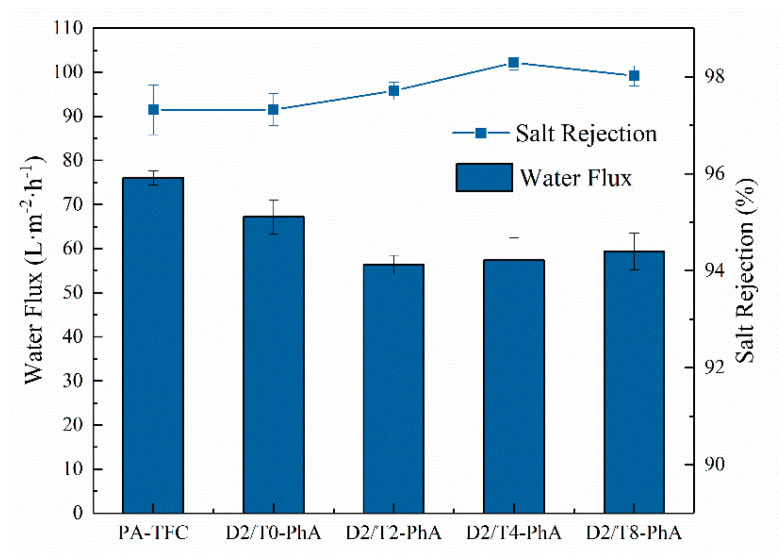
The permselectivity of the PA-TFC membrane and the DA/TA-PhA membranes.

The separation performance of the PA-TFC membrane and the modified membranes were evaluated using their water flux and salt rejection amount (Figure 4). The flux and salt rejection of the PA-TFC membrane were 76.05 L·m^−2^·h^−1^ and 97.32%, respectively. The flux of the modified membrane was decreased; for example, the flux of D2/T4-PhA dropped to 57.37 L·m^−2^·h^−1^. The modified layer of DA/TA-PhA on the surface of the membrane led to an increase in the water permeation resistance during the separation process. The formation of the modified layer also increased the transport resistance of salt ions. Taking D2/T4-PhA as an example, the salt rejection rate increased from 97.32% to 98.29%. All changes in membrane flux and rejection rate were consistent with the trade-off effect. Water/salt permeability selectivity as a function of diffusive water permeability for the PA-TFC membrane and the D2/T4-PhA modified membrane is shown in Figure S2 [39,40,41,42].

### 3.6. Membrane Anti-Fouling Properties

**Figure 5:**
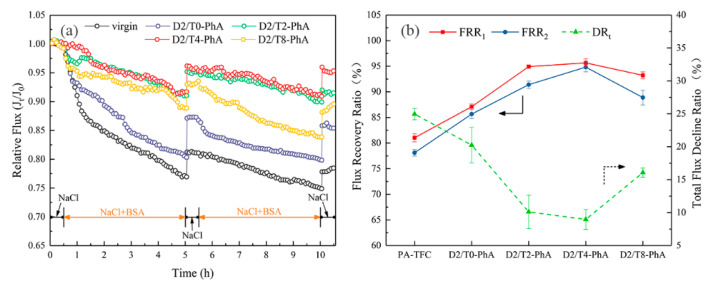
(**a**) Time-dependent relative water flux and (**b**) *FRR_i_* values and *DR_t_* values of PA-TFC and DA/TA-PhA membranes tested with 2000 mg·L^−1^NaCl and 100 mg·L^−1^ BSA aqueous solution.

**Figure 6:**
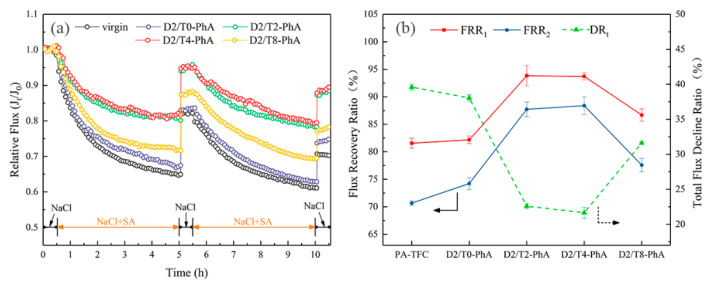
(**a**) Time-dependent relative water flux and (**b**) *FRR_i_* values and *DR_t_* values of PA-TFC and DA/TA-PhA membranes tested with 2000 mg·L^−1^ NaCl and 100 mg·L^−1^ SA aqueous solution.

**Figure 7:**
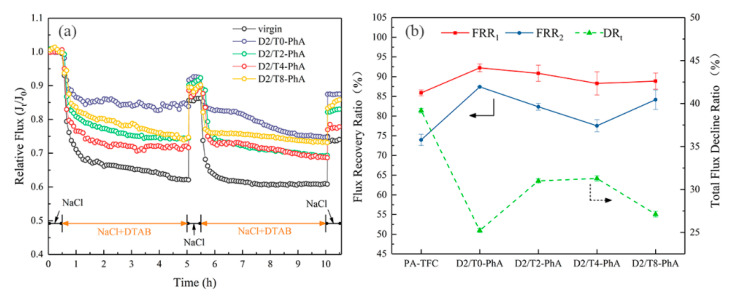
(**a**) Time-dependent relative water flux and (**b**) *FRR_i_* values and *DR_t_* values of PA-TFC and DA/TA-PhA membranes tested with 2000 mg·L^−1^ NaCl and 50 mg·L^−1^ DTAB aqueous solution.

The anti-fouling performance of the membrane was evaluated by three model foulants (BSA, SA, and DTAB). The results of BSA, SA, and DTAB fouling tests were listed in Figure 5, Figure 6 and Figure 7, respectively. The raw data of BSA, SA and DTAB fouling tests were listed in Tables S1–S3, respectively. BSA (100 mg·L^−1^, negatively charged) is used to represent the protein in the water treatment process. SA (100 mg·L^−1^, negatively charged) is used as a model foulant to characterize the polysaccharides foulant in wastewater. DTAB (50 mg·L^−1^, positively charged) is used as a model foulant to characterize the foulant surfactants in wastewater.

As shown in Figure 5, Figure 6 and Figure 7, in each cycle, all the flux of the membrane decrease as the time goes on. After rinsing, the flux could be recovered to some extent. It can be clearly seen that the modified membranes had better anti-fouling performance than the PA-TFC membrane. The *FRR_i_* values first increased and then decreased with the concentration of TA increased. In all experiments, the *FRR*_2_ values were always less than the *FRR*_1_ values, which might be due to the irreversible adsorption of foulants on the membrane surface.

However, the decline rate was different in the three polluted environments due to their different adhesion mechanisms. In Figure 5a, the D2/T4-PhA membrane showed the best anti-fouling performance. As shown in Figure 5b, the *FRR*_1_, *FRR*_2_, and *DR_t_* values of the D2/T4-PhA membrane were 95.7%, 94.8%, and 9.0%, respectively, much higher than those of PA-TFC. In Figure 6a, the decline curve of membrane flux was relatively smooth, and the decline rate of membrane flux gradually decreased. As shown in Figure 6b, the *FRR*_1_, *FRR*_2_, and *DR_t_* values of the D2/T4-PhA membrane reached 93.7%, 88.4%, and 21.7%, respectively. Under the same conditions, the *FRR*_1_, *FRR*_2_, and *DR_t_* values of PA-TFC were 81.6%, 70.7%, and 39.6%, respectively. The higher *FRR_i_* values and lower *DR_t_* value indicate that the modified membrane has better anti-fouling performance. This may be due to the increase in the density of surface negative charges and the increase in surface hydrophilicity, which reduced the adsorption of the two negatively charged foulants on the membrane surface.

In Figure 7a, the anti-fouling performances of the modified membranes were also better than that of the PA-TFC membrane. As the TA content of the interlayer increased, the content of PhA successfully loaded on the membrane increased. Thus, the negative charge density on the surface of the membrane increased, resulting in the worsening of the anti-fouling performance. Because DTAB was positively charged, the negatively charged modified membrane surface would adsorb more foulants.

**Figure 8:**
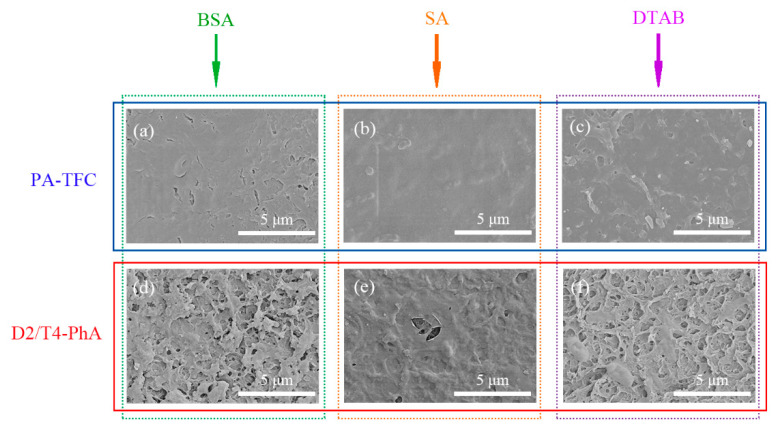
SEM surface images of (**a**–**c**) PA-TFC and (**d**–**f**) D2/T4-PhA fouled by (**a**,**d**) BSA, (**b**,**e**) SA, or (**c**,**f**) DTAB, respectively.

The membrane surface after anti-fouling tests was observed using SEM. It could be found that the contaminants attached to the modified membrane were significantly less than that of the PA-TFC membrane (Figure 8). After the BSA or SA fouling test, the surface of the contaminated PA-TFC was completely covered by contaminants, while the ridge and valley structure of the polyamide were still partly observed on the contaminated D2/T4-PhA membrane. After the DTAB fouling tests, there is no obvious difference between the SEM images of the D2/T4-PhA membrane and the PA-TFC membrane. That is because the low DTAB concentration in the feed liquid, and the negative charge on the surface of D2/T4-PhA would increase the electrostatic attraction between the membrane surface with DTAB [43]. Overall, the modified membrane had a better anti-fouling performance.

The improved anti-fouling ability of DA/TA-PhA modified membranes against BSA, SA, or DTAB foulants could be attributed to their excellent surface properties, including enhanced hydrophilicity, higher negative charge density, and reduced roughness. Because of their weak hydrophobic properties, the BSA or SA foulants were hardly adsorbed onto the hydrophilic membrane surface. Additionally, the surfaces of the modified membranes were smoother and thus could reduce the possibility of foulant molecules depositing and accumulating in the valley structures.

### 3.7. Membrane Stability Assessment

**Figure 9:**
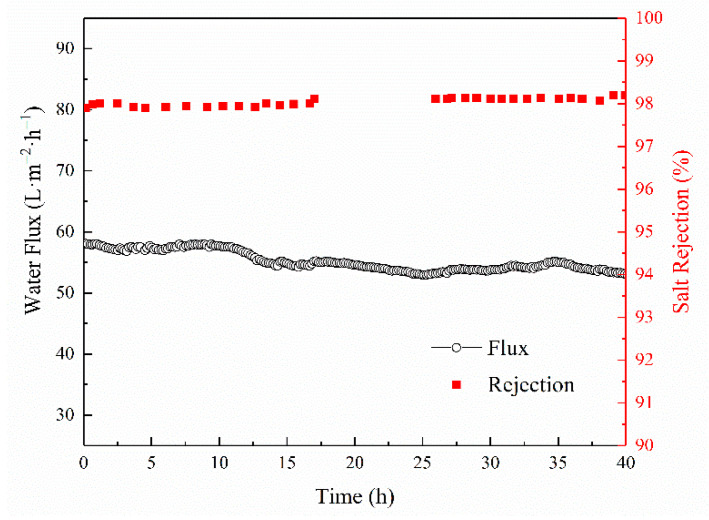
Operational stability of D2/T4-PhA.

The stability of D2/T4-PhA was tested in 2000 mg·L^−1^ NaCl solution for 40 h. It can be seen from Figure 9 that the modified membrane showed good stability in 40 h. The rejection rate was almost unchanged, maintaining a rate around 98%. There were some fluctuations and a slight decrease of membrane flux in long-term operation. Overall, the membrane maintained good stability during the stability test.

The D2/T4-PhA modified membrane was soaked in a pH = 4 HCl solution or a pH = 10 NaOH solution for 60 min. After taking it out, the changes in water flux and salt rejection were measured. The results are listed in Figure S3. The strong chemical cleaning will affect the permeability of the modified membrane to a certain extent. At the same time, the permeability of the modified membrane after alkaline cleaning decreases more than that of acid cleaning.

## 4. Conclusions

A simple and versatile strategy for preparing anti-fouling RO membranes was developed via introducing a DA/TA interlayer and a hydrophilic PhA modified layer onto the RO membrane. The DA/TA interlayer with strong adhesion to the RO membrane surface could easily bind Fe^3+^-PhA via the complexation between Fe^3+^ and TA. After the modification, the hydrophilicity and wettability were significantly improved, and the anti-fouling performance was also enhanced. Compared to the PA-TFC membrane with a water flux of 76.05 L·m^−2^·h^−1^ and a salt rejection of 97.32%, the modified D2/T4-PhA membrane has a water flux of 57.37 L·m^−2^·h^−1^ and an enhanced salt rejection (98.29%). In the 100 mg·L^−1^ BSA anti-fouling experiment, the *FRR*_1_, *FRR*_2_, and *DR_t_* values of the D2/T4-PhA membrane were 95.7%, 94.8%, and 9.0% respectively, while the values for the PA-TFC membrane were 81.0%, 78.1%, and 25.0% respectively. For the SA and DTAB contaminants, the modified membranes also exhibited improved anti-fouling performance. Additionally, the DA/TA-PhA modified membrane also showed good operation stability. Therefore, the integration of a DA/TA interlayer and PhA modification onto the membrane surface could be an effective strategy for preparing efficient anti-fouling RO membranes.

## Figures and Tables

**Table 1:** The relative surface atomic concentration of the PA-TFC membrane and the DA/TA-PhA modified membranes tested by EDS spectra.

Samples	Atomic Percent (%)					Atomic Ratio	
	C	N	O	P	Fe	N/C	O/C
PA-TFC	84.09	5.70	10.20	-	-	0.068	0.121
D2/T0-PhA	79.50	7.11	11.83	1.53	0.03	0.089	0.149
D2/T2-PhA	77.72	8.34	12.26	1.59	0.09	0.107	0.158
D2/T4-PhA	72.89	8.18	16.56	2.24	0.12	0.112	0.227
D2/T8-PhA	77.58	8.56	12.35	1.43	0.07	0.110	0.159

## Supplementary Materials

The following are available online at https://www.mdpi.com/article/10.3390/membranes11050342/s1, Figure S1: Characterization of the thickness of (a) PA-TFC membrane and (b) D2/T4-PhA modified membrane; Figure S2: Water/salt permeability selectivity as a function of diffusive water permeability for the PA-TFC membrane and the D2/T4-PhA modified membrane; Figure S3: Changes in water flux and salt rejection before and after chemical cleaning; Table S1: Experimental data of BSA anti-fouling experiment; Table S2. Experimental data of SA anti-fouling experiment; Table S3. Experimental data of DTAB anti-fouling experiment.
