# Supplementary material for: A Dopamine/Tannic-Acid-Based Co-Deposition Combined with Phytic Acid Modification to Enhance the Anti-Fouling Property of RO Membrane
Source: Membranes (Basel). 2021 May 6;11(5):342. doi: 10.3390/membranes11050342 (PMC8148169; doi:10.3390/membranes11050342)
Supplement: Supplementary file 1 [file membranes-11-00342-s001.zip › membranes-1205341-supplementary.pdf]

Supplementary Materials:

A Dopamine/Tannic-Acid-Based Co-Deposition Combined with Phytic Acid Modification to Enhance the Anti-Fouling Property of RO Membrane

Lixin Xie, Yan Liu, Wen Zhang * and Shichang Xu *

School of Chemical Engineering and Technology, Tianjin Key Laboratory of Membrane Science and Desalination Technology, State Key Laboratory of Chemical Engineering (Tianjin University), Tianjin University, Tianjin 300350, China; xie_lixin@tju.edu.cn (L.X.); liuyan_ly1128@163.com (Y.L.)
* Correspondence: zhang_wen@tju.edu.cn (W.Z.); xushichang@sina.com (S.X.)

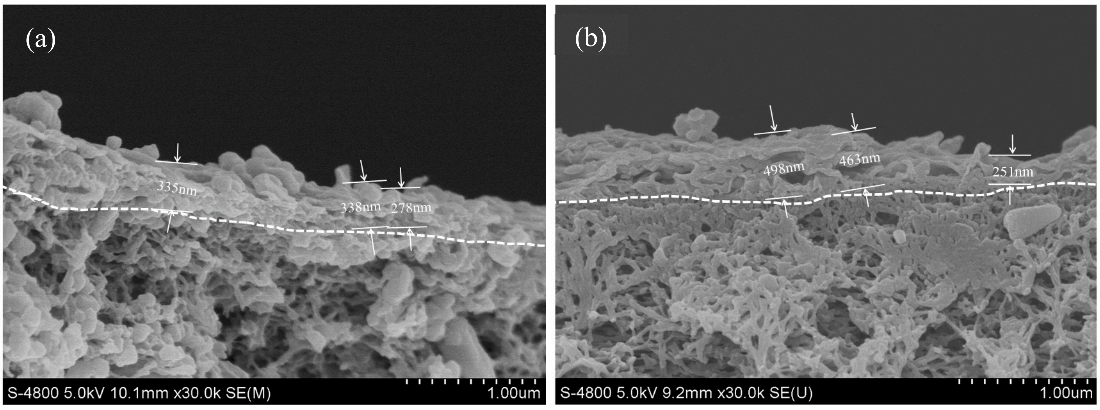

Figure S1. Characterization of the thickness of (a) PA-TFC membrane and (b) D2/T4-PhA modified membrane.

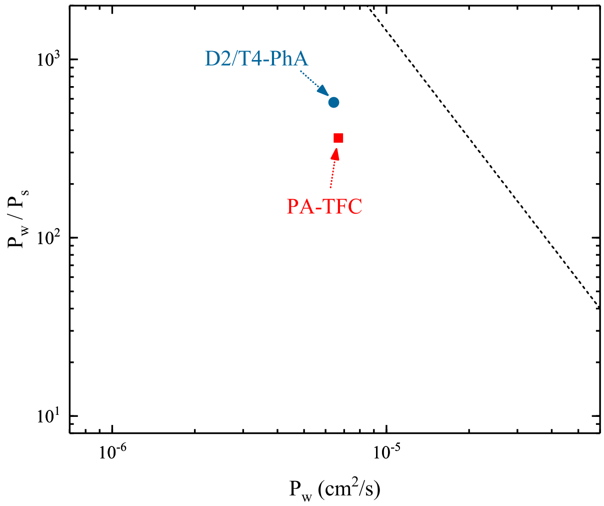

Figure S2. Water/salt permeability selectivity as a function of diffusive water permeability for the PA-TFC membrane and the D2/T4-PhA modified membrane.

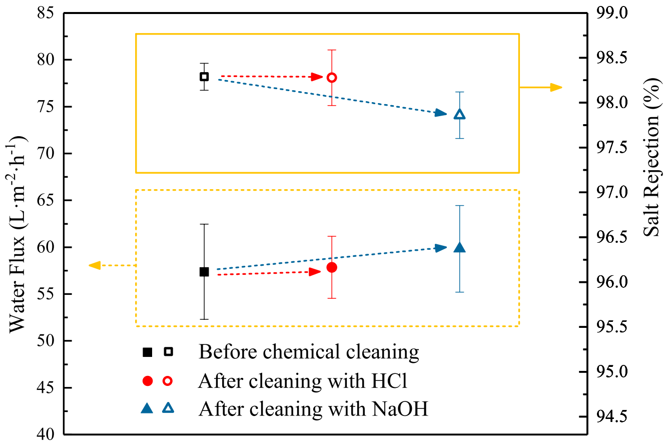

Figure S3. Changes in water flux and salt rejection before and after chemical cleaning.

Table S1. Experimental data of BSA anti-fouling experiment.

T (h)	Real-Time Water Flux (L·m⁻²·h⁻¹)				
	PA-TFC	D2/T0-PhA	D2/T2-PhA	D2/T4-PhA	D2/T8-PhA
0.06778	63.67801	62.52452	61.39685	59.14019	60.18454
0.15222	63.66782	62.51083	61.73539	59.19988	60.56924
0.23667	63.62136	62.593	61.6526	59.15792	60.24585
0.32139	63.48387	62.22261	61.29136	59.16146	59.9471
0.40583	63.57426	61.88957	61.30424	59.31631	59.74754
0.49028	63.69265	62.09936	61.23555	59.15319	59.64054
0.57472	62.48202	61.65614	60.92522	59.13605	58.60905
0.65944	61.67749	60.85498	59.80411	58.71526	57.76992
0.74389	60.64636	59.07214	59.63975	58.96821	57.5415
0.82833	59.62159	58.20686	59.5318	59.11241	57.58057
0.91306	58.87434	58.08672	59.38584	58.81218	57.42729
0.9975	57.93932	58.03132	59.23006	58.69517	57.27521
1.08194	57.00494	57.7238	59.4901	58.87187	56.74083
1.16639	56.24496	57.29241	59.86605	58.73476	56.74083
1.25111	55.88343	57.27747	59.84888	58.40203	56.93499
1.33556	55.5098	57.12434	59.76486	58.40439	57.00231
1.42	55.2081	56.98676	59.7391	58.0492	56.85444
1.50444	55.09353	56.86662	59.76363	57.32582	56.6669
1.58917	54.76255	56.66369	59.25091	57.05514	57.0474
1.67361	54.68044	56.69979	59.40608	56.97299	56.85685
1.75806	54.60788	56.50993	59.26073	56.91448	56.85685
1.84278	54.41948	56.17253	59.15646	56.76141	56.85685
1.92722	54.01466	56.11713	58.89888	56.70231	56.62903
2.01167	54.04522	55.60668	58.90685	56.91744	56.67051
2.09611	53.85681	55.43674	58.88784	56.58116	56.63925
2.18083	53.56593	55.28236	58.5677	56.37372	56.75646
2.26528	53.59394	55.04145	58.86576	56.44523	56.70597
2.34972	53.40808	54.719	58.80382	56.42454	56.2858
2.43444	53.3317	54.70717	58.61676	56.25197	56.21006
2.51889	52.89315	54.36853	58.3604	56.21356	56.09766
2.60333	52.99499	54.35981	58.47018	56.26675	56.02012
2.68778	52.77031	54.30441	58.47264	56.2106	55.90651
2.7725	52.65701	53.58293	58.22732	55.92574	56.01951
2.85694	52.33239	53.20757	58.23406	56.11131	56.02132
2.94139	52.46924	52.89943	58.32483	56.0924	56.05077
3.02583	52.1459	52.64358	58.15863	55.9943	55.44486
3.11056	52.13126	52.59191	58.0237	55.75021	55.6829
3.195	52.01987	52.41637	58.05559	55.64088	55.40098
3.27972	51.94349	52.40018	57.96973	55.72007	55.51279
3.36417	51.58514	52.19538	57.83174	55.52859	55.43525
3.44861	51.64434	52.16114	57.83358	55.7248	55.09683

3.53306	51.60678	52.14247	57.69988	55.91628	55.58853
3.61778	51.60615	51.7976	57.63916	55.83827	55.31983
3.70222	51.21088	51.72975	57.29817	55.53509	55.59093
3.78667	51.30636	51.62828	57.47357	55.64147	55.30601
3.87111	51.1205	51.59342	57.20249	55.50672	55.47372
3.95583	50.7997	51.32886	57.22212	55.29514	55.20623
4.04028	50.78252	51.32824	56.86272	55.25673	55.36251
4.12472	50.66922	50.93731	56.80691	55.26914	55.4112
4.20944	50.40634	50.90432	56.80753	55.13321	55.39738
4.29389	50.20203	50.90307	56.54442	54.94291	55.28437
4.37833	50.07027	50.80036	56.3776	54.88558	55.02049
4.46278	50.03208	50.69951	56.38987	54.68168	54.74638
4.5475	49.64127	50.66528	56.28622	54.27094	54.56004
4.63194	49.65655	50.66279	55.95136	53.93525	54.33583
4.71639	49.35676	50.53642	56.10039	54.0174	54.04731
4.80111	49.27974	50.49969	55.97528	54.096	53.47205
4.88556	48.96849	50.38017	56.27211	54.0635	53.57063
4.97	49.10407	50.14673	55.79374	54.16279	53.41795
5.05444	48.96849	49.99484	55.83054	54.20297	53.41795
5.0675	51.71563	54.23967	58.38616	56.84593	55.8999
5.15194	51.62588	54.35048	58.30214	56.81283	56.01591
5.23639	51.77227	54.33367	58.16905	56.56934	55.83137
5.32111	51.65643	54.34985	58.36224	56.47005	55.90831
5.40556	51.61569	54.32558	58.34262	56.64676	56.02192
5.49	51.60933	54.01184	58.35182	56.37076	56.24072
5.57472	51.26116	53.76844	58.03597	56.27798	55.50618
5.65917	51.51449	53.14594	58.23958	56.56579	55.36191
5.74361	51.44257	52.76248	58.11324	56.66508	55.43525
5.82806	51.20452	52.53464	57.98322	56.37372	55.19781
5.91278	51.45721	52.42259	57.96912	56.27502	55.06737
5.99722	51.20452	52.36346	57.98935	56.37372	54.85098
6.08167	51.12877	52.24518	57.7471	56.56934	54.65442
6.16639	51.10713	52.18853	57.74771	56.66508	54.24867
6.25083	50.82453	52.13251	57.99242	56.64676	54.32321
6.33528	50.92509	52.13251	57.69742	56.71827	54.17895
6.41972	50.8398	52.08458	57.9268	56.27502	54.12485
6.50444	50.71314	51.90654	57.73422	55.78567	53.88441
6.58889	50.70359	51.79387	57.61524	55.98366	53.90184
6.67333	50.6724	51.79387	57.30859	55.78863	53.86878
6.75778	50.58966	51.7422	57.58151	55.9807	54.0425
6.8425	50.51391	51.68369	57.5441	56.17573	54.00463
6.92694	50.41462	51.56977	57.68577	55.98366	53.99381
7.01139	50.20203	51.5679	57.68516	55.98366	53.46905
7.09611	50.3166	51.40045	57.5533	56.0794	53.34101

7.18056	50.03527	51.28715	57.59439	55.9807	53.22801
7.265	50.11865	51.22926	57.53183	56.0794	53.00921
7.34944	50.03463	51.22926	57.33926	56.03921	52.83188
7.43417	49.92006	51.17324	57.38648	55.98366	52.70325
7.51861	49.88314	51.00392	57.40549	55.78863	52.58844
7.60306	49.68455	51.00392	57.26075	55.59005	52.50068
7.68778	49.66037	51.00392	57.12522	55.78863	52.24761
7.77222	49.47324	50.94727	57.29142	55.59655	52.08291
7.85667	49.44714	50.94665	57.03015	55.40152	52.15324
7.94111	49.30456	50.89124	57.09332	55.29928	51.99094
8.02583	49.4325	50.89124	56.99029	55.19999	51.82925
8.11028	49.12252	50.89	56.90565	55.10425	51.8587
8.19472	49.03087	50.72192	56.74129	55.20354	51.8046
8.27917	49.08306	50.66528	56.79281	55.46358	51.68378
8.36389	48.84947	50.66528	56.77318	55.00792	51.62908
8.44833	48.83865	50.60863	56.65727	54.80993	51.5876
8.53278	48.6356	50.73188	56.47082	54.71714	51.57017
8.6175	48.64069	50.50156	56.19913	54.51561	51.32432
8.70194	48.62096	50.44989	56.57263	54.69587	51.45837
8.78639	48.59487	50.44989	56.71921	54.7136	51.37001
8.87083	48.40837	50.39324	56.71001	54.53984	51.13858
8.95556	48.45675	50.38826	56.37699	54.61786	51.10252
9.04	48.6827	50.33037	56.46898	54.32354	51.05804
9.12444	48.55922	50.27186	56.45059	54.41928	51.0412
9.20917	48.54967	50.27186	56.09487	54.77802	50.85306
9.29361	48.37846	50.22143	56.21998	54.41928	50.8771
9.37806	48.47266	50.16416	56.17889	54.22425	50.94984
9.4625	48.21042	50.15669	56.07402	54.41928	50.73705
9.5475	48.27534	50.05025	55.80539	54.12851	50.71601
9.63194	48.08948	49.99298	55.5613	54.2278	50.82361
9.71639	47.98192	49.99298	55.49813	53.93643	50.82601
9.80083	47.89535	49.93571	55.23012	53.9453	50.55612
9.88556	47.86162	49.87844	55.17247	53.83124	50.42688
9.97	47.74196	49.7639	55.40246	53.93348	50.40163
10.05444	47.66876	49.69231	55.14978	53.73845	50.40584
10.0675	49.52479	53.41548	56.42421	56.72595	52.98035
10.15194	49.5407	53.46217	55.97466	56.3288	53.08675
10.23667	49.54834	53.70245	56.17828	56.27798	53.19194
10.32111	49.69219	53.30281	56.23593	56.20292	53.50511
10.40556	49.88251	53.16586	55.97773	56.18342	53.68003
10.49	49.93597	53.1559	56.1145	56.33708	53.85495

Table S2. Experimental data of SA anti-fouling experiment.

T (h)	Real-Time Water Flux (L·m^{-2}·h^{-1})				
	PA-TFC	D2/T0-PhA	D2/T2-PhA	D2/T4-PhA	D2/T8-PhA
0.0675	61.55043	60.88486	60.08838	58.76954	59.5944
0.15222	61.47233	61.0464	59.89916	58.30799	59.17181
0.23667	61.51784	61.03486	59.94168	58.46728	59.37358
0.32111	61.48401	60.98385	59.8291	58.31907	59.79677
0.40583	61.44527	60.31096	60.11593	58.29282	60.13841
0.49028	60.55352	60.46643	59.85725	59.00352	59.25811
0.57472	57.75957	59.95509	58.81294	58.69835	58.4046
0.65917	55.96931	57.64856	57.66743	57.31487	56.7303
0.74389	54.44042	56.05075	57.0351	57.00445	55.75417
0.82833	53.36786	54.64789	56.25486	55.23819	54.69471
0.91278	52.36172	53.42175	55.64648	54.63486	53.74954
0.99722	51.4835	52.21505	54.90098	54.08812	52.88233
1.08194	50.40663	51.1462	54.52134	53.6254	52.10024
1.16639	50.07269	50.26197	54.0429	53.05007	51.47468
1.25083	49.35129	49.14332	53.43092	52.56752	50.83068
1.33556	48.46815	48.66416	53.08841	52.09021	50.20155
1.42	48.03458	48.17104	52.7423	51.59074	49.50516
1.50444	47.53151	47.65908	52.43751	51.53355	48.95937
1.58889	46.93557	46.90664	51.91836	51.43786	48.47368
1.67361	46.34148	47.16474	51.83033	50.97573	47.84337
1.75806	45.96326	46.82526	51.57105	50.70265	47.58445
1.8425	45.61701	46.05277	51.18303	50.38464	47.09222
1.92722	45.09119	45.98111	51.13692	50.7044	46.65892
2.01167	44.8335	45.87301	50.92016	50.51243	46.33334
2.09611	44.5832	45.53292	50.79081	50.38464	46.10657
2.18056	44.29968	45.14486	50.4483	50.09289	45.69231
2.26528	43.90239	45.007	50.44351	49.86241	45.53637
2.34972	43.79846	44.85518	50.26986	49.74629	45.34234
2.43417	43.48481	44.44282	50.09621	49.46621	45.15663
2.51889	43.24619	44.26792	49.71357	49.40028	44.99533
2.60333	43.07645	44.02075	49.83453	49.0175	44.9257
2.68778	42.85628	43.95273	49.66387	48.93815	44.83225
2.77222	42.63734	43.67945	49.41058	48.70008	44.4543
2.85694	42.33906	43.94726	49.35908	48.67149	44.39121
2.94139	42.26096	43.68127	49.36028	48.63239	44.26443
3.02583	42.16563	43.66487	49.27166	48.62364	44.2555
3.11028	42.10352	43.40069	49.06507	48.32489	44.08289
3.195	41.83907	43.32417	49.27226	48.93931	43.85731
3.27944	41.82062	43.20514	49.18364	48.78468	44.00195
3.36389	41.63304	42.94826	48.93513	48.47076	43.76268
3.44861	41.40119	42.927	49.01238	48.44042	43.79422

3.53306	41.41349	42.98652	48.79501	48.28638	43.24783
3.6175	41.22468	42.6774	48.79561	48.01096	43.50912
3.70194	41.09922	42.31545	48.58963	48.05648	43.3871
3.78667	40.93194	42.47517	48.66507	47.94094	43.44067
3.87111	41.00574	41.99783	48.62136	47.74956	43.33175
3.95556	40.9424	42.06767	48.70819	47.28392	43.28652
4.04028	40.68164	42.00998	48.63274	47.21157	43.23414
4.12472	40.69209	41.89581	48.79501	47.63986	43.17878
4.20917	40.66134	41.96564	48.7501	47.55175	43.30378
4.29361	40.56663	41.57637	48.58963	47.67078	43.18295
4.37833	40.33785	41.48345	48.66507	47.47881	43.25318
4.46278	40.34708	41.52171	48.7501	47.28567	43.19545
4.54722	40.3151	41.47252	48.70879	47.01551	43.14426
4.63167	40.22162	41.2964	48.45969	47.5535	43.22045
4.71639	40.02543	41.147	48.70759	47.63402	43.11807
4.80083	40.06418	40.91016	48.57705	47.557	43.05022
4.88528	40.00268	40.59011	48.33035	47.6667	42.67108
4.97	39.77513	40.75408	48.49202	47.90127	42.74548
5.05444	39.93995	40.9806	47.98424	47.67312	42.64429
5.06778	50.74796	50.44659	56.29678	55.3053	52.00143
5.15222	50.52164	50.44963	56.38361	55.58304	52.07583
5.23667	50.74796	50.34335	56.96684	55.3053	51.89311
5.32111	50.36481	50.68769	57.13929	55.68807	52.40736
5.40583	50.9097	50.65125	56.94528	55.38815	52.33058
5.49028	50.52102	50.76542	57.40037	55.3053	52.56806
5.57472	49.05179	50.78971	56.87822	55.49202	52.2294
5.65944	48.93801	49.70143	56.05367	54.69262	52.0681
5.74389	48.30825	49.23928	55.389	54.18789	51.48956
5.82833	47.76951	48.48987	55.10158	53.53613	50.99436
5.91278	47.40051	47.93601	54.58062	53.19945	50.65807
5.9975	46.89744	47.69066	54.1902	52.85518	50.06525
6.08194	46.39622	46.99591	53.48721	52.81667	49.67242
6.16639	45.54936	46.70562	53.27823	53.07341	49.14566
6.25111	45.26769	46.15298	52.97584	52.74023	48.78914
6.33556	44.7966	45.95803	52.44949	52.53659	48.41952
6.42	44.35626	45.39871	52.32434	52.15148	48.12668
6.50444	43.86119	45.06652	51.89081	51.80488	47.70349
6.58917	43.66562	44.81753	51.62973	51.51488	47.48744
6.67361	43.31999	44.49444	51.28243	51.38243	47.26067
6.75806	42.82922	44.07358	51.1968	51.23013	46.83926
6.8425	42.6933	43.7924	51.02255	50.90279	46.76844
6.92722	42.37965	43.66001	50.41776	50.73066	46.45298
7.01167	42.12812	43.2428	50.45908	50.73066	46.27561
7.09611	41.76588	42.94522	50.15728	50.49901	45.97444

7.18083	41.65826	42.67133	49.93872	50.21309	45.8679
7.26528	41.40611	42.46788	49.98543	50.34613	45.50364
7.34972	41.14043	42.18913	49.89621	50.1139	45.26198
7.43417	41.03096	41.97475	49.76567	49.44754	45.05307
7.51889	40.90673	41.85694	49.33693	49.92193	44.89356
7.60333	40.68533	41.49377	49.37585	50.03979	44.79832
7.68778	40.51497	41.31219	49.28902	49.84724	44.68583
7.7725	40.40243	41.16097	49.11597	49.40961	44.57631
7.85694	40.24683	41.04012	48.90519	49.4242	44.3674
7.94139	40.12076	40.8306	48.89741	49.27016	44.07516
8.02583	39.88952	40.70185	48.8543	48.88621	44.01683
8.11056	39.86922	40.59922	48.81178	48.64348	43.6722
8.195	39.74499	40.42857	48.55909	48.54195	43.69363
8.27944	39.483	40.20265	48.59502	48.42525	43.39603
8.36417	39.52359	40.07391	48.5513	47.99288	43.31985
8.44861	39.36738	40.00224	48.29981	48.00396	43.04486
8.53306	39.2739	39.76054	48.42136	47.88901	42.96332
8.6175	39.01437	39.74779	48.2052	47.77231	42.70143
8.70222	39.11708	39.51337	48.24771	47.41871	42.6062
8.78667	38.99162	39.49272	47.86747	47.46364	42.46812
8.87111	38.7327	39.28502	48.07466	47.31018	42.29372
8.95556	38.83602	39.10648	47.85729	47.31135	42.23242
9.04028	38.7407	39.15202	47.65131	47.07386	42.07766
9.12472	38.61462	38.99716	47.76987	47.15789	41.97827
9.20917	38.42028	38.91396	47.59741	47.15672	41.78542
9.29389	38.4578	38.78339	47.59741	46.92099	41.80804
9.37833	38.36432	38.74392	47.34891	47.00443	41.68781
9.46278	38.11278	38.62246	47.46688	46.92682	41.60508
9.54722	37.88831	38.47731	47.33574	46.85038	41.49675
9.63194	37.89323	38.43723	47.25011	46.65258	41.43366
9.71639	37.82988	38.45424	47.00281	46.69692	41.3658
9.80083	37.60725	38.24897	47.12017	46.58197	41.36104
9.88528	37.70504	38.23986	47.12017	46.2698	41.33188
9.97	37.61094	38.21678	46.91658	46.23596	41.24676
10.05444	37.58019	38.18156	46.90281	46.38825	41.34973
10.06778	43.48542	44.78291	52.23991	51.27623	45.8554
10.15222	43.34459	45.06287	52.1968	51.72144	46.10419
10.23667	43.36427	45.03919	52.63811	51.64559	46.06372
10.32139	43.32737	45.18373	52.76745	51.62049	46.23811
10.40583	43.30277	45.17401	52.71536	51.87432	46.34763
10.49028	43.22097	45.44365	52.98422	52.22033	46.58154

Table S3. Experimental data of DTAB anti-fouling experiment.

T (h)	Real-time water flux ($L{\cdot}m^{-2}{\cdot}h^{-1}$)				
	PA-TFC	D2/T0-PhA	D2/T2-PhA	D2/T4-PhA	D2/T8-PhA
0.06778	63.08946	61.48238	60.42293	58.81695	59.68355
0.15222	63.00587	61.1898	59.99225	58.88174	59.98979
0.23667	62.92982	61.1292	60.01561	58.87585	60.17022
0.32139	62.80098	61.12247	60.00123	58.87585	59.77079
0.40583	62.78024	61.15797	60.00423	58.99424	59.31261
0.49028	62.52758	61.34527	60.04855	59.27048	59.20993
0.57472	58.49261	60.06599	59.4795	55.99741	55.62697
0.65944	49.89662	56.05	51.96445	48.87051	56.06498
0.74389	47.86467	54.49526	50.25969	46.95567	51.94431
0.82833	46.34748	54.25042	49.45404	46.55927	50.27182
0.91278	45.6291	53.47122	49.02096	45.9261	49.88011
0.9975	44.69766	52.97664	48.44832	45.07617	49.64153
1.08194	44.03145	52.853	48.20093	45.06439	48.87651
1.16639	43.49283	52.60449	47.91701	44.95778	48.76671
1.25111	42.84924	52.19805	47.61331	44.75988	48.55958
1.33556	42.65315	51.68511	47.30543	43.72442	48.48242
1.42	43.00008	52.34006	47.26829	43.83161	48.16549
1.50444	42.46146	51.97035	47.13711	43.41519	47.96845
1.58917	42.15915	52.19805	46.80886	43.51473	47.81295
1.67361	42.29554	52.17969	46.81305	43.07475	47.6355
1.75806	42.51111	52.46187	46.55308	43.33568	47.3981
1.84278	42.22766	52.19928	46.59621	43.11892	47.6355
1.92722	42.00014	52.16439	46.29072	42.95636	47.63372
2.01167	41.56396	52.67549	46.31588	42.94281	47.59751
2.09611	41.77514	52.62285	46.22902	42.62534	47.261
2.18083	41.78708	52.48268	46.04453	42.40741	47.36189
2.26528	41.73806	52.67549	45.93432	42.38503	47.26041
2.34972	41.72612	52.42759	45.92533	42.80263	47.25566
2.43417	41.64378	52.16439	45.81691	42.80263	46.91083
2.51889	41.4898	51.77448	45.47069	42.58293	46.82537
2.60333	41.43261	51.48985	45.49345	42.75904	46.49479
2.68778	41.38421	51.649	45.47069	42.58588	46.31852
2.7725	41.3465	51.65206	45.36227	42.60532	46.47342
2.85694	41.28114	51.66797	45.08254	42.4239	46.39627
2.94139	41.18875	51.77325	45.12507	42.44629	46.41764
3.02583	41.13847	51.38641	45.03761	42.40859	46.77136
3.11056	40.91975	51.49046	44.97472	42.21127	46.61349
3.195	40.9914	51.65083	44.95196	41.59695	46.2384
3.27944	40.95809	52.07808	44.90823	41.75362	45.78912
3.36417	40.79342	51.83446	45.07775	42.03104	45.90366
3.44861	40.72743	51.42191	44.565	41.71475	45.80514
3.53306	40.7312	51.29582	44.71236	42.38621	45.70544
3.6175	40.66458	51.46965	44.67043	42.46808	45.49593
3.70222	40.46597	51.35152	44.67282	42.52462	45.43243
3.78667	40.41758	51.15626	44.71295	42.4239	45.34993
3.87111	40.51562	50.6574	44.71295	42.42744	45.23716
3.95556	40.28811	50.57415	44.77825	42.52462	45.08107
4.04028	40.1071	50.70208	44.49971	42.42862	45.15348

4.12472	40.14104	51.76162	44.73512	42.18771	44.90184
4.20917	40.04299	51.40722	44.67043	42.1459	44.79026
4.29389	39.89404	51.35213	44.69139	42.42331	44.87988
4.37833	39.42329	51.14524	44.45658	42.16415	44.8591
4.46278	39.29131	51.77509	44.69139	42.48398	44.80153
4.54722	39.29256	51.82957	44.56261	42.54877	44.27807
4.63194	39.0971	51.47333	44.45658	42.23248	44.78254
4.71639	39.17818	50.81654	44.58297	41.95624	44.22584
4.80083	39.08013	51.79162	44.53984	41.93386	44.06381
4.88556	39.04682	51.95138	44.60513	42.4292	43.87567
4.97	39.09333	51.70347	44.47815	42.46808	44.15343
5.05444	39.01602	51.33254	44.60394	42.19066	44.32139
5.0675	53.83103	56.09774	54.05196	52.17362	53.13843
5.15194	53.77635	56.34625	54.23346	51.09281	53.09748
5.23667	53.73298	56.70005	54.3305	51.46623	52.31287
5.32111	54.14339	56.69454	54.67912	52.05759	52.84999
5.40556	54.17984	56.60946	54.88517	52.64953	53.30045
5.49	54.21504	56.45582	55.2907	52.82211	53.54023
5.57472	46.3481	52.24763	53.30561	51.63115	48.74119
5.65917	42.8373	51.37784	53.08817	49.21743	45.76894
5.74361	41.61361	51.00813	47.52346	44.17736	45.45082
5.82833	40.63692	50.9004	45.51142	43.54713	45.19028
5.91278	40.29376	50.7284	44.94357	43.07416	45.07336
5.99722	39.9506	50.56558	44.55122	42.91749	45.11609
6.08167	39.60116	50.66903	44.17565	42.64419	45.07336
6.16639	39.39061	50.5815	44.15768	43.07416	44.97365
6.25083	39.28125	50.53436	43.98517	42.87802	45.15645
6.33528	39.11784	50.47744	43.74138	43.1148	45.02054
6.41972	39.01539	50.49335	43.85399	42.80145	44.99442
6.50444	38.83627	50.48111	43.85339	43.07534	44.97543
6.58889	38.8721	50.30177	43.72221	42.99641	45.07751
6.67333	38.88467	50.42174	43.52394	42.84092	44.97662
6.75806	38.78599	50.3691	43.59043	43.07593	45.01816
6.8425	38.75897	50.10957	43.45985	43.07475	44.91667
6.92694	38.69423	50.03611	43.26278	42.95636	44.98018
7.01139	38.67223	49.89043	43.28674	42.80145	44.83774
7.09611	38.54088	49.65049	43.15555	42.83915	44.79857
7.18056	38.5943	49.43503	43.02437	42.83679	44.86029
7.265	38.48117	49.28935	42.8279	42.56585	44.78492
7.34972	38.2128	49.13021	42.93752	42.56173	44.7588
7.43417	38.35107	48.74887	42.85006	42.48163	44.64307
7.51861	38.25302	48.63991	42.76321	42.32554	44.68343
7.60306	38.15309	48.5279	42.56734	42.05519	44.62111
7.68778	38.11915	48.4116	42.71828	42.16828	44.46561
7.77222	38.21971	47.98619	42.5889	42.08817	44.56532
7.85667	38.18892	47.90784	42.48048	42.08876	44.46799
7.94111	38.05002	47.73095	42.54398	41.89793	44.42466
8.02583	38.18766	47.52834	42.54338	42.0493	44.42585
8.11028	38.18829	47.34961	42.45772	42.00984	44.46739
8.19472	38.07139	47.3239	42.26304	41.74066	44.22821
8.27944	38.08773	47.07539	42.45652	41.85316	44.31071

8.36389	38.23605	46.94623	42.36907	41.77247	44.14928
8.44833	38.17195	46.78342	42.17619	41.69413	44.19142
8.53278	38.13612	46.70507	42.28341	41.42614	44.03117
8.6175	38.05442	46.5753	42.19536	41.45735	43.95283
8.70194	38.30205	46.46512	42.1079	41.33838	44.03295
8.78639	38.25428	46.45655	42.04561	41.15107	43.89704
8.87111	38.13172	46.45472	42.06358	41.22117	43.8733
8.95556	38.0563	46.13398	42.02165	41.14165	43.79615
9.04	38.28508	46.07766	41.93479	41.02444	43.75757
9.12444	38.18452	46.10215	41.74132	40.83655	43.75638
9.20917	38.26371	45.9883	41.84734	40.94434	43.71721
9.29361	38.3209	46.02257	41.76108	40.78825	43.65845
9.37806	38.25177	46.01401	41.56761	40.866	43.67982
9.46278	38.21657	46.10215	41.67243	40.56149	43.5997
9.54722	38.24988	45.92158	41.6287	40.67045	43.46082
9.63167	38.25365	45.90015	41.54005	40.78766	43.63946
9.71611	38.2348	45.872	41.3933	40.48315	43.51957
9.80083	38.15184	45.85425	41.4538	40.6304	43.60088
9.88528	38.23731	45.72693	41.45499	40.51083	43.44301
9.96972	38.31965	45.73978	41.43702	40.51024	43.52254
10.05417	38.21846	45.75509	41.54125	40.44369	43.40444
10.0675	47.11362	53.53794	49.20486	45.33121	49.45101
10.15222	46.21863	53.55324	49.25457	46.2365	49.74242
10.23667	46.41661	53.4755	49.40492	45.59096	49.90623
10.32111	46.26828	53.47979	49.5996	45.68991	50.52644
10.40556	46.31731	53.55508	49.68645	45.58742	50.78401
10.49028	46.54608	53.55508	49.72898	45.82773	50.93654
